# High peak viraemia followed by spontaneous HIV‐1 control in women living with HIV‐1 subtype A1 in East Africa

**DOI:** 10.1002/jia2.70016

**Published:** 2025-08-10

**Authors:** Yifan Li, Bethany L. Dearlove, Eric Lewitus, Hongjun Bai, Shida Shangguan, Phuc Pham, Meera Bose, Eric Sanders‐Buell, Shana Howell Miller, Yvonne Rosario, Philip K. Ehrenberg, Sodsai Tovanabutra, Rasmi Thomas, Julie A. Ake, Sandhya Vasan, Leigh Anne Eller, Sorachai Nitayaphan, Lucas Maganga, Hannah Kibuuka, Fredrick K. Sawe, Merlin L. Robb, Morgane Rolland

**Affiliations:** ^1^ U.S. Military HIV Research Program, CIDR, Walter Reed Army Institute of Research Silver Spring Maryland USA; ^2^ Henry M. Jackson Foundation for the Advancement of Military Medicine Bethesda Maryland USA; ^3^ Armed Forces Research Institute of Medical Sciences Bangkok Thailand; ^4^ National Institute for Medical Research‐Mbeya Medical Research Center Mbeya Tanzania; ^5^ Makerere University Walter Reed Project Kampala Uganda; ^6^ Kenya Medical Research Institute/U.S. Army Medical Research Directorate‐Africa/Kenya‐Henry Jackson Foundation MRI Kericho Kenya

**Keywords:** acute infection, HIV‐1 subtype A1, HIV‐1, peak viraemia, persons living with HIV‐1, viraemic controllers

## Abstract

**Introduction:**

Cases of spontaneous control of HIV‐1 can help define strategies to induce remission. Since the identification of viral control in the absence of treatment typically occurs after a prolonged period post‐HIV‐1 diagnosis, our knowledge of the early events after HIV‐1 acquisition that led to viral control is limited.

**Methods:**

The RV217 prospective cohort enrolled 2276 participants in East Africa (Kenya, Uganda, Tanzania) and Thailand between 2009 and 2015. We analysed HIV‐1 sequences and clinical data from 102 individuals who were diagnosed with acute HIV‐1 infection and had a negative HIV‐1 RNA test in the week before. We focused on 69 participants with longitudinal follow‐up and identified viraemic controllers who maintained viral loads <2000 copies/ml for over a year without treatment. We evaluated viral genetic and clinical features that are associated with viral control.

**Results:**

Eleven women from East Africa showed control of viral replication for an average duration of 891 (range: 405–1425) days within an average of 130 days from diagnosis. The majority were living with subtype A1 (*n* = 6), or A1 recombinant strains (*n* = 4), with one living with subtype D; 10 were from Kenya, one from Uganda. Controllers had significantly slower CD4+ T cell decline (*p* = 0.028) and higher Natural Killer (NK) cell counts (*p* = 0.047) than non‐controllers, but none carried human leukocyte antigen (HLA) alleles previously reported to be associated with viral control. Peak viraemia was recorded at an average of 541 million copies/ml with no difference between controllers and non‐controllers (*p* = 0.97). Viral loads became lower in controllers (3459 copies/ml) than in non‐controllers (23,157 copies/ml) as early as nadir viraemia (*p* = 0.009), with a more significant difference observed at set point (1069 vs. 24,084 copies/ml, respectively; *p*<0.0001).

**Conclusions:**

Our findings confirm the role of HIV‐1 subtype A1 in mediating viral control. The fact that controllers showed high viral loads in acute infection indicates that these viruses were not intrinsically impaired for replication, underlining the intersection between host immunity and favourable genotypes in the subsequent control of HIV‐1. These data suggest that conducting HIV‐1 remission studies in East Africa could provide favourable conditions to achieve durable post‐treatment control of viraemia.

## 1 . INTRODUCTION

While spontaneous control of HIV‐1 is rare, characterizing those events can help define strategies to induce remission. Among people living with HIV‐1 (PLWH), a small fraction (<1%), denoted elite controllers, can suppress HIV‐1 replication to <50 RNA copies/ml in the absence of antiretroviral treatment (ART) [1]. Protective HLA alleles are overrepresented among elite controllers [2], in agreement with the fact that the major histocompatibility complex locus was the only significant hit in genome‐wide association studies designed to identify polymorphisms associated with viral load (VL) control [3]. Besides elite controllers, viraemic controllers were defined by their ability to maintain VL <2000 copies/ml for at least 12 months [4].

Controllers have been identified in different cohorts [5], but the events leading to control are rarely known. Yet, the prevailing impression seems to be that control of viraemia is seen early after acquisition. As such, Goulder and Deeks [6] stated that “although data from the acute phase of peak viremia are scarce, the collective data suggest that peak viremia during the acute phase is likely lower than that in more typical infection. Elite control is likely driven in part by a favorable host response that is active during the earliest stages of the infection.”

To evaluate whether the initial stages after HIV‐1 acquisition relate to subsequent viral control, we considered 102 incident cases identified among 2276 participants who underwent twice weekly HIV‐1 RNA testing in the prospective RV217 cohort [7]. We showed that viraemic controllers had peak viraemia levels that were as high as those seen in non‐controllers and that control occurred after the acute phase, predominantly in individuals carrying HIV‐1 subtype A1.

## 2 METHODS

### 2.1 Study design

The RV217 acute infection cohort enrolled 2276 seronegative individuals in Uganda, Kenya, Tanzania and Thailand between 2009 and 2015 [7]. All participants signed written informed consent, and the protocols were approved by local and US Institutional Review Boards. Investigators adhered to U.S. Army (AR 70‐25) and host nation regulations for the protection of human subjects. HIV‐1 RNA testing was performed twice weekly. Plasma HIV‐1 RNA levels and T‐cell immunophenotypes (CD4/CD8/NK/B cells) were measured longitudinally up to 2115 days post‐diagnosis before the initiation of ART. Individuals who maintained HIV‐1 RNA levels between 50 and 2000 copies/ml were defined as viraemic controllers [4]. Samples were tested for the presence of ART.

### 2.2 HIV‐1 sequence analysis

HIV‐1 near full‐length genome sequences were obtained via single genome amplification from plasma samples, and some characteristics for this dataset were previously described [8, 9, 10]. *env* alignments were edited manually. Consensus sequences were derived from sequences sampled in the first month after diagnosis. Pairwise diversity and divergence from the consensus were estimated at 1 week, 1 month and 6 months after diagnosis using Hamming distances. HIV‐1 subtypes were determined using RIP (https://www.hiv.lanl.gov/content/sequence/RIP/RIP.html), REGA (https://www.genomedetective.com/app/typingtool/hiv) and comet (https://comet.lih.lu/) based on one randomly sampled *env* sequence from the earliest time point. Phylogenies were reconstructed using IQ‐TREE [11].

### 2.3 Heritability of set point VL

Sequences sampled from RV217 participants (this manuscript and [8]) were analysed together with sequences sampled from placebo recipients in the RV144 vaccine efficacy trial (https://clinicaltrials.gov/study/NCT00223080) [12, 13]. Trees were reconstructed as above. The maximum likelihood ancestral states of set point viral load (SPVL) were estimated using fastAnc in the R‐package phytools [14]. A phylogenetic Ornstein‐Uhlenbeck mixed model (POUMM) was fitted to the phylogeny and associated SPVL data to estimate the heritability of SPVL. The analysis was implemented using the R‐package POUMM with 10^6^ iterations for each Markov Chain Monte Carlo (MCMC) chain [15].

### 2.4 HLA genotyping

HLA genotyping was performed as described previously [16, 17].

### 2.5 Data analysis

Mann‐Whitney U tests were used for pairwise comparisons. Spearman's rho was calculated for testing the correlation between two variables. SPVL was defined as the average VL between day 42 and 1 year after diagnosis. The slope of T‐cell counts was calculated from day 42 and 1 year, with at least two measurements using individual linear regression. Participants with more than 1 year of VL data without treatment (*n* = 69) were included for the characterization of viraemic controllers and non‐controllers. Fisher's exact test was used to evaluate the association of HLA allele presence/absence and viral control, and the Benjamini‐Hochberg procedure was used to adjust *p*‐values. Data visualization and statistical analysis were performed in R with the packages tidyverse, ggpubr and ggtree [18].

## 3 RESULTS

### 3.1 Eleven women from East Africa maintained VLs <2000 copies/ml in the RV217 cohort

**Figure 1:**
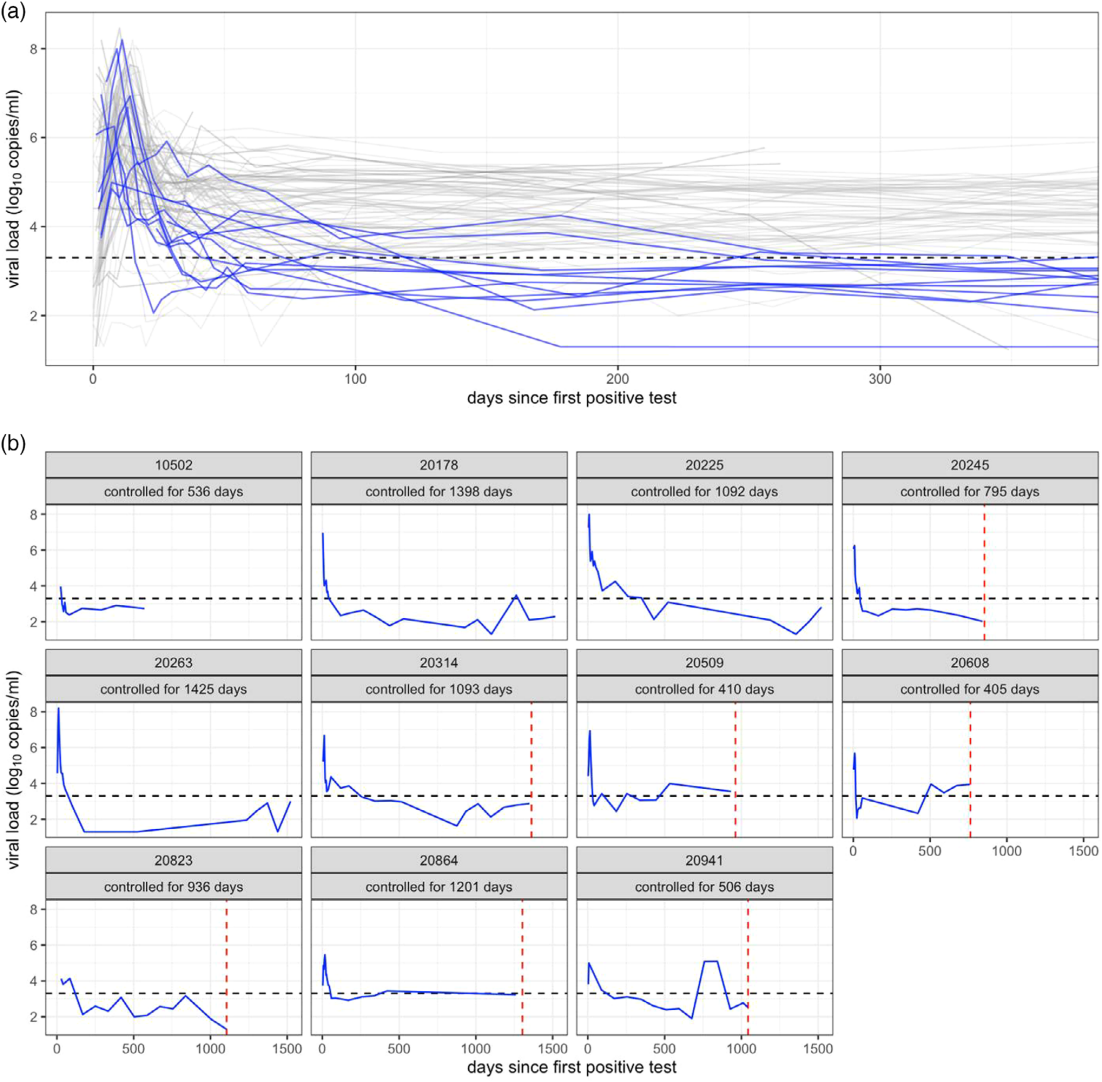
Rapid control of viral loads in 11 participants. (A) Viral trajectories during the first year after HIV‐1 diagnosis for 102 RV217 participants who were ART‐naive. Blue lines indicate viraemic controllers. (B) Viral trajectory of the 11 viraemic controllers (the participant ID is shown above each VL plot). Red dashed lines indicate ART initiation; black horizontal lines indicate VL = 2000 copies/ml.

We investigated the viral trajectory of 102 PLWH (including 59 females) who were diagnosed during acute HIV‐1 infection (*n* = 72 in Fiebig stage I‐II) (flowchart at https://www.hivresearch.org/publication‐supplements). Participants were from East Africa (Kenya = 28, Tanzania = 20, Uganda = 13; 57 females) and Thailand (*n* = 41; 23 males, 2 females, 16 transgender women). Peak viral RNA was recorded on average at 6.8 log_10_ copies/ml (range: 4.47−8.46) and occurred on average 12 days (range: 0–41) after the first positive RNA test (Figure 1A). Subsequently, a rapid decline in viraemia led to an average SPVL of 4.25 log_10_ copies/ml (range: 2.43−5.96).

We identified 11 viraemic controllers who maintained <2000 HIV‐1 RNA copies/ml for at least 1 year in the absence of ART (Figure 1B). The average duration of viraemic control was 891 days (range: 405–1425) before the participants started ART or exited the study. The viraemic controllers were women from East Africa (10 from Kenya and one from Uganda). While our small cohort size did not allow for powered evaluations of HLA associations with viral control, the distribution of HLA alleles did not differ between viraemic controllers and non‐controllers (*p*>0.4). We also checked that, among the viraemic controllers with HLA typing (*n* = 10), none had protective HLA alleles such as B*27, B*57 or B*58:01, which have been previously associated with viral control [3, 19, 20].

### 3.2 Low set point VL was associated with HIV‐1 subtype A1

**Figure 2:**
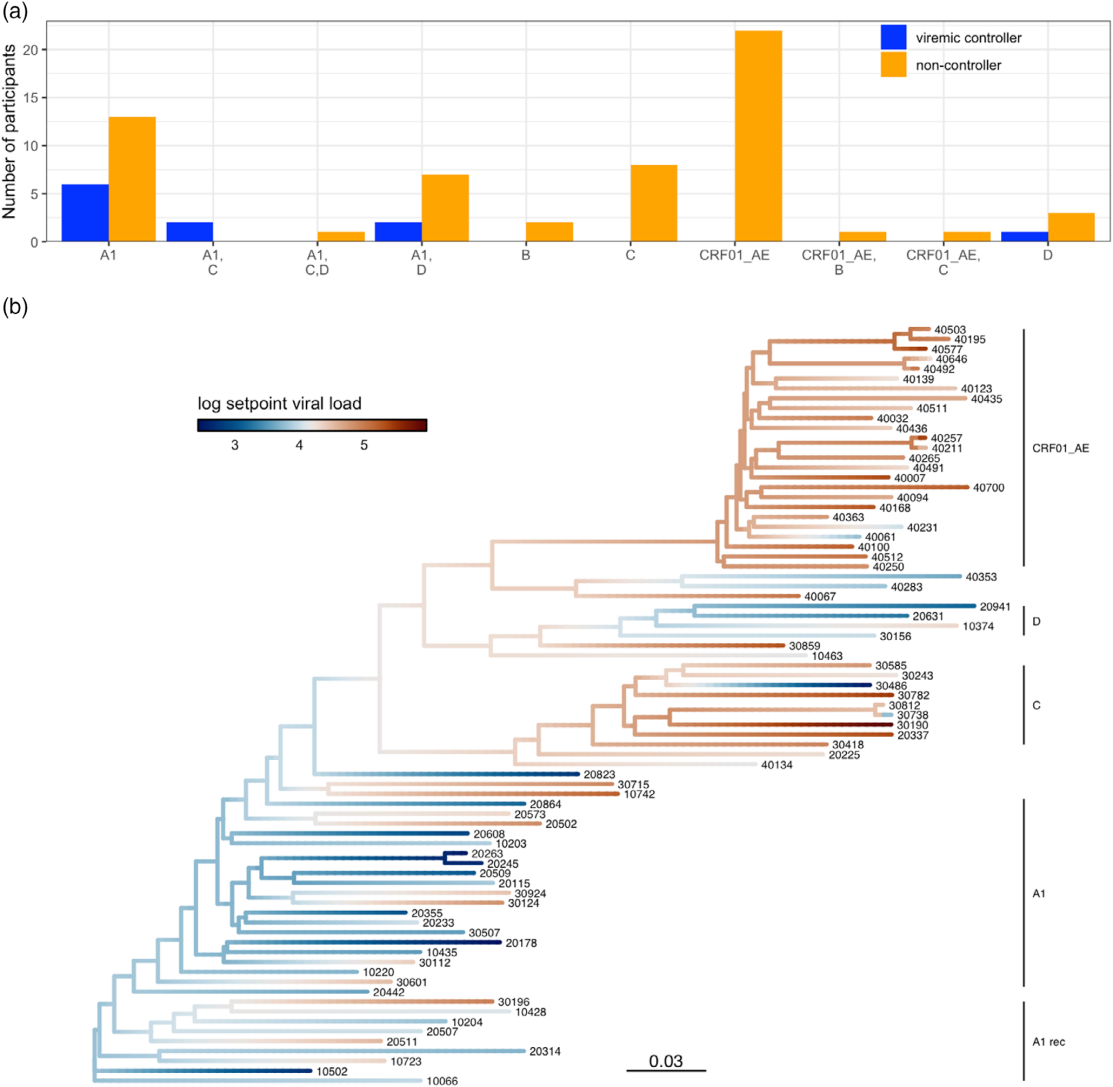
HIV‐1 subtype A1 associated with viraemic control. (A) Distribution of subtypes using *env* sequences in viraemic controllers (in blue) and non‐controllers (in orange). (B) Phylogeny reconstructed from consensus sequences for RV217 participants with branches colour‐coded to show the reconstructed ancestral estimates of SPVL; subtypes are denoted on the right of the figure (“A1rec” corresponds to the different recombinants including subtype A1 with either subtypes C and D or both).

We analysed 1761 *genome* and 238 *envelope (env)* sequences sampled from 93 participants in the first 6 months post‐diagnosis. We previously reported that, across the cohort, 21% of the infections were established by multiple founder variants [8]. We found that all viraemic controllers had a single founder lineage, which is consistent with prior studies showing that individuals with multiple HIV‐1 founder variants have a higher SPVL than individuals with single founders [21]. We also found subtype‐specific differences between controllers and non‐controllers (Figure 2). The majority of viraemic controllers had subtype A1 viruses (*n* = 6 or 5, considering either *env* or *genome* sequences, respectively) or A1 recombinant strains (*n* = 4 or 5, considering either *env* or *genome* sequences, respectively), while one had subtype D (Figure 2A). When we compared SPVL across individuals, those with subtype A1—both controllers and non‐controllers—had significantly lower SPVL than individuals with subtype C (East Africa) or CRF01_AE (Thailand) (*p*<0.0064). Among East Africans, A1 or A1 recombinant viruses were associated with viral control (*p* = 0.05). The phylogenetic reconstruction highlights the A1 clade, which groups participants with low SPVL (Figure 2B). The analysis of the heritability of SPVL on the phylogeny reconstructed from participants’ sequences was hampered by the small size of our cohort and showed little to no evidence that SPVL was heritable (*H* ^2^ = 0.12 [95% HPD: 7.19e‐09, 0.48]).

### 3.3 Viraemic control occurred after the acute phase

**Figure 3:**
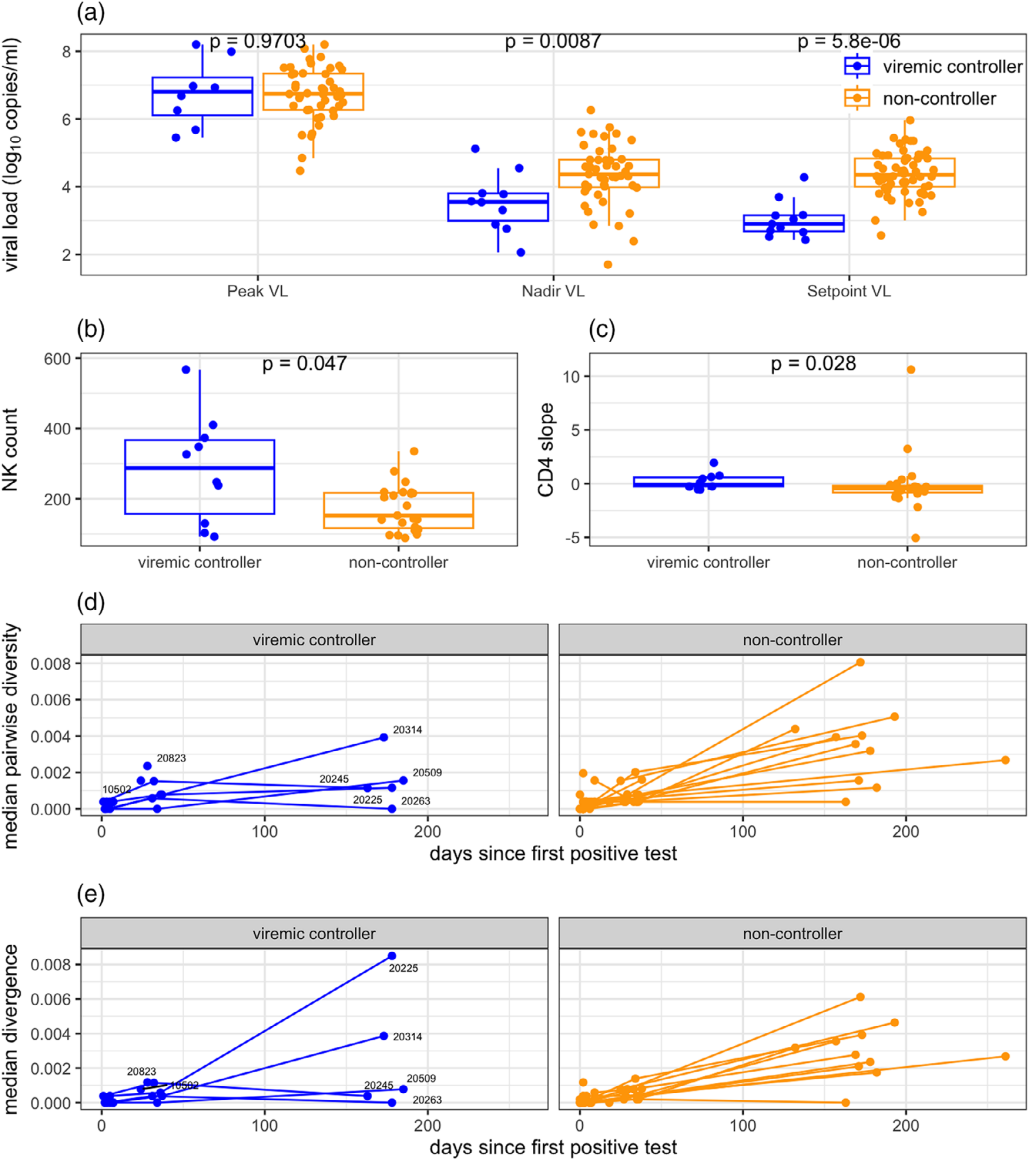
Similar viral loads in viraemic controllers and non‐controllers in acute HIV‐1 infection. (A) Comparison of peak VL, nadir VL and set point VL in viraemic controllers (blue) and non‐controllers (orange) during the first year after diagnosis. (B) Average NK cell counts and (C) CD4+ T cell slope during the first year after diagnosis in viraemic controllers and non‐controllers with single founders in East Africa. (D) Env median pairwise diversity and (E) divergence from the consensus based on nucleotide sequences sampled within 42 days of infection in viraemic controllers and non‐controllers with single founders in East Africa. *p* values of Wilcoxon rank sum tests are shown.

While SPVL were lower in controllers than in non‐controllers (*p*<0.0001), both groups had comparable high peak VL (*p* = 0.97) (Figure 3A). Strikingly, one viraemic controller (pid: 20263) who had the second highest peak VL recorded in our cohort, at 158 million copies/ml on day 11, subsequently controlled viraemia with 617 copies/ml 97 days after HIV‐1 diagnosis and undetectable levels on day 178. Viraemia remained under 1000 copies/ml until her last follow‐up visit at day 1522 (Figure 1B). When including all participants, there was only a moderate correlation between peak viraemia and SPVL (Spearman's rho = 0.35, *p* = 0.006). While there was no difference between controllers and non‐controllers at peak viraemia or VL downslope after peak viraemia, VL were lower among controllers compared to non‐controllers (*p* = 0.009) when nadir VL were measured a median of 36 (range: 18–42) days after diagnosis, foretelling the hierarchy seen with SPVL (nadir VL and SPVL are correlated).

During the first year, controllers showed higher natural killer cell counts (*p* = 0.047) than non‐controllers with single founder variants in East Africa (Figure 3B), with a slower decline in CD4+ T cell counts (*p* = 0.028) (Figure 3C). There were no significant differences in CD8+ T‐ or B‐cell counts or slopes between the two groups (*p*≥0.096). The results remained consistent when including participants with multiple founder variants. Lastly, although low‐level diversity was more common among controllers, there were no significant differences in *env* pairwise diversity (*p*≥0.052) or divergence from the consensus at baseline (*p*≥0.53) between controllers and non‐controllers with infections established by a single founder variant in East Africa (*n* = 35) (Figures 3D and E). Similarly, both groups had participants with sequences with no shared mutations at 6 months.

## 4 DISCUSSION

Prolonged control of viral replication in the absence of ART is rare, and identifying the early steps post‐HIV‐1 acquisition that led to viral control is challenging, as retrospective data are often missing when control is identified. By comparing VL kinetics in 102 PLWH, we identified 11 controllers who maintained VL <2000 copies/ml for at least a year. These participants did not present known protective HLA alleles [2, 3, 19, 20]. While new HLA alleles associated with control in East Africans remain to be discovered, given the limited number of studies conducted in Africa, the small size of our cohort precluded such discovery investigations.

Since HLA alleles that associate with control are overrepresented among controllers, it seems logical to conclude that viruses in controllers are weak or impaired viruses that are easier to control from the earliest days in acute infection [6]. This is consistent with the observation that HLA alleles mediating viral control are associated with cytotoxic T lymphocyte (CTL) escape mutations that incur a fitness cost [22, 23]; at the population level, CTL escape mutations typically correspond to mutations away from the consensus (representative of the most fit virus) to a rare residue. Our findings show that viruses found in controllers are not necessarily weak viruses. By following participants from the initial days of detectable viraemia until spontaneous viral control in the ensuing months, we showed that viruses from viraemic controllers replicated to similar levels as those found in non‐controllers in the first weeks of acute infection, with very high values when peak viraemia was measured (>100 million copies/ml). These results demonstrate that viruses associated with later control were not impaired in replication. Nonetheless, the process leading to viraemic control starts early, as nadir VL measured about 5 weeks after diagnosis were significantly lower among controllers than non‐controllers. Together, our data support that the initial stages of viral replication are governed by viral demographic processes [24] before the advent of effective host immunity, particularly the CTL response, ushers in the control of viraemia starting in the first weeks of infection.

Notwithstanding the importance of host immunity in the control of viraemia, our results also illustrate the role of the subtype: subtype A1 is associated with viral control, as previously observed [25, 26]. While our study participants were mostly females in East Africa and males in Thailand, the results remain consistent when including East African participants only. The different sex distribution across geographic regions is a limitation, as sex‐based comparisons are confounded by multiple other factors. Investigating the interplay between the host immune response and HIV‐1 genetics in the setting of viraemic control is critically needed and may inform the design of cure strategies. Importantly, our data suggest that HIV‐1 remission studies should be conducted in East African women where subtype A1 is prevalent. We hypothesize that the proportion of post‐treatment controllers would be higher than in cohorts where subtype B or CRF01_AE are prevalent.

## 5 CONCLUSIONS

By following participants since their HIV‐1 diagnosis in acute infection, we identified 11 viraemic controllers. These participants carried HIV‐1 subtype A1 viruses, supporting a role for viral genotypes in controlling viraemia. Yet, these viruses were not replication defective, as very high peak VL were recorded. Viral control was achieved rapidly after peak viraemia, possibly reflecting the onset of host‐mediated immune responses.

## COMPETING INTERESTS

The investigators have adhered to the policies for protection of human participants as prescribed in AR 70–25. Material has been reviewed by the Walter Reed Army Institute of Research. There is no objection to its presentation and/or publication.

## AUTHORS’ CONTRIBUTIONS

YL, BLD and MR designed research; YL, BLD, EL, HB, SS and MR performed research; PP, MB, ES‐B, SHM and ST sequenced HIV‐1; YR, PKE and RT contributed HLA genotyping data; JAA and SV contributed resources; LAE, SN, LM, HK, FKS and MLR conducted the cohort study; YL, BLD and MR analysed data; YL and MR wrote the manuscript.

## FUNDING

This work was supported by a cooperative agreement (WW81XWH‐18‐2‐0040) between the Henry M. Jackson Foundation for the Advancement of Military Medicine, Inc. and the U.S. Department of Defense (DOD).

## DISCLAIMER

The opinions or assertions contained herein are the private views of the author, and are not to be construed as official, or as reflecting true views of the Department of the Army or the Department of Defense, or the Department of Health and Human Services.

## Data Availability

Sequences were previously deposited in GenBank under accession KY580473‐KY580727, MN791130‐MN792579, MW443137‐MW443225, OM824463‐OM826780, ON959609‐ON959788 and PV865314‐PV865327. Data files and sequence alignments generated during this study are available at https://www.hivresearch.org/publication‐supplements.
